# Patient experience of severe acute dyspnoea and relief during treatment in ambulances: a prospective observational study

**DOI:** 10.1186/s13049-020-0715-2

**Published:** 2020-04-03

**Authors:** Tim Alex Lindskou, Ulla Møller Weinreich, Kenneth Lübcke, Torben Anders Kløjgaard, Birgitte Schantz Laursen, Søren Mikkelsen, Erika Frischknecht Christensen

**Affiliations:** 1 Department of Clinical Medicine, Centre for Prehospital and Emergency Research, Aalborg University, Søndre Skovvej 15, 9000 Aalborg, Denmark; 2 Department of Respiratory Diseases, Aalborg University Hospital, Mølleparkvej 4, Aalborg, Denmark; 3 Emergency Medical Services, North Denmark Region, Hjulmagervej 20, 9000 Aalborg, Denmark; 4 Clinical Nursing Research Unit, Aalborg University Hospital, Søndre Skovvej 15, 9000 Aalborg, Denmark; 5 Department of Regional Health Research, University of Southern Denmark, J. B. Winsløws Vej 19, 5000 Odense, Denmark

**Keywords:** Acute dyspnoea, Prehospital, Ambulance, Verbal numerical rating scale, Dyspnoea score, Vital signs, Respiratory rate, Blood oxygen saturation, Blood pressure, Heart rate

## Abstract

**Background:**

Acute dyspnoea is common among ambulance patients, but little is known of the patients’ experience of symptom. We aimed to investigate ambulance patients initial perceived intensity of acute dyspnoea, and whether they experienced relief during prehospital treatment. Furthermore, to investigate the validity and feasibility of using a subjective dyspnoea score in the ambulance, and its association with objectively measured vital signs.

**Methods:**

We performed a prospective observational study in the North Denmark Region from 1. July 2017 to 30. March 2019. We studied patients over the age of 18 to whom an ambulance was dispatched. Patients with acute dyspnoea assessed either at the emergency call or by ambulance professionals on scene were included. Patients were asked to assess dyspnoea on a 0 to 10 verbal numeric rating scale at the primary contact with the ambulance personnel and immediately before release at the scene or arrival at the hospital. Patients received usual prehospital medical treatment. We used visual inspection and Wilcoxon matched-pairs signed-ranks test, to assess dyspnoea scores and change hereof. Scatterplots and linear regression analyses were used to assess associations between the dyspnoea score and vital signs.

**Results:**

We included 3199 patients with at least one dyspnoea score. Of these, 2219 (69%) had two registered dyspnoea scores. The initial median dyspnoea score for all patients was median 8 (interquartile range 6–10). In 1676 (76%) of patients with two scores, the first score decreased from 8 (6–9) to 4 (2–5) during prehospital treatment. The score was unchanged for 370 (17%) and increased for 51 (2%) patients. Higher respiratory rate, blood pressure, and heart rate was seen with higher dyspnoea scores whereas blood oxygen saturation lowered.

**Conclusions:**

We found that acute dyspnoea scored by ambulance patients, was high on a verbal numerical rating scale but decreased before arrival at hospital, suggesting relief of symptoms. The acute dyspnoea score was statistically associated with vital signs, but of limited clinical relevance; this stresses the importance of patients’ experience of symptoms. To this end, the dyspnoea scale appears feasible in the prehospital setting.

## Background

Acute dyspnoea is a distressing symptom with a sensation of difficult, laboured and uncomfortable breathing [1, 2]. It is the symptom of numerous acute as well as chronic diseases, and it is a frequent symptom seen among patients in the emergency departments as well as in the ambulances [3, 4]. A study of emergency medical service patients from the United States of America showed that in 2002–2006, one in eight of the patients had respiratory distress, when excluding traumatic injury and cardiac arrest [5]. European studies have showed that dyspnoea was the main cause for calling an ambulance in 6–9% of the emergency calls [6–8].

Dyspnoea may be affected by and may induce factors such as fear and anxiety, and like pain, is difficult to assess objectively [9, 10]. Studies have reported conflicting results regarding consistent correlation between objective measurements, e.g. oxygen saturation, and the degree of dyspnoea [11–13]. Furthermore, previous studies have suggested there is poor correlation between health care professionals’ and patient’s assessment of dyspnoea intensity [14, 15].

Our aim was to investigate the intensity of dyspnoea experienced by ambulance patients, whether they experience relief of symptoms before arrival to hospital, the feasibility of an acute dyspnoea scale, also in ambulances, and validate possible associations between dyspnoea intensity and objectively measured vital signs.

Several scoring systems that quantify the intensity and quality of dyspnoea exists [1, 9, 16]. These include both unidimensional tools, used for measuring the severity of dyspnoea, and multidimensional tools that incorporate several other aspects, such as anxiety and quality of life [17]. However, there is no consensus on which scoring system should be considered the gold standard when assessing acute dyspnoea [9, 18].

A previous review of subjective dyspnoea rating scales relating to emergency medicine have suggested the use of unidimensional scales in a verbal numerical tool to assess the severity of breathlessness [19]. Verbal numerical scales have the advantage of requiring no physical tools; they are easy for patients to use and require less explanation [20]. They are well known primarily for the assessment of pain, and have also been used and validated in the assessment of dyspnoea in the emergency departments, yet assessment of dyspnoea have not been studied in the prehospital setting [20–22].

## Method

Our primary aim was to investigate the initial intensity of dyspnoea experienced by the patients in ambulances and if the patients experienced relief of dyspnoea before arrival to hospital. Our secondary aim was to investigate the feasibility of the prehospital use of a dyspnoea scale and possible associations between the reported dyspnoea score and the objectively measured vital signs.

### Study design

This prospective observational cohort study was carried out in the North Denmark region.

### Setting

The region is home to 589,731 of Denmark’s total population of 5,814,461 citizens [23]. The population is geographically widespread compared to Denmark’s other regions and its 7885 km^2^ covers both urban and rural areas [24]. The common national emergency calls are answered by the police. If the call concerns healthcare, the emergency call is forwarded to a health care professional at an Emergency Medical Coordination Centre. At these centres, health care professionals assess the urgency and determines the appropriate response, e.g. if an ambulance dispatched with or without lights and sirens [25].

The health care professionals use a criteria-based dispatch guideline, Danish Index for Emergency Care (DI) as a support tool to determine the emergency response. The DI is categorising the callers’ concern within 37 groups of symptoms or tentative diagnoses including respiratory insufficiency [18].

The Danish prehospital professionals encompass different levels; basic life-support paramedics, paramedics with special competencies, and prehospital emergency physicians (anaesthesiologists). On the ambulance level, the treatment options include oxygen, continuous positive airway pressure and medications for acute asthma, acute exacerbation of chronic obstructive lung disease (beta-2-agonist and continuous positive airway pressure), and pulmonary oedema/acute heart failure (diuretics, nitro-glycerine spray). In the most severe cases attended by the prehospital emergency physicians, treatment at a critical care level is applied, including usual medication for medical emergencies as well as endotracheal intubation and mechanical ventilation.

Patient data are recorded online as ambulance professionals enter data on observations of vital signs and treatment into a portable tablet computer, which directly transfer data to an electronic prehospital medical record (*Amphi Systems A/S, Aalborg, Denmark*). Vital signs, blood oxygen saturation, blood pressure and heart rate, are measured automatically by a monitor/defibrillator (LIFEPAK 15) [26]. Respiratory rate is only measured automatically when monitoring end-tidal CO2 using capnography, otherwise it is measured manually. Measurements, alongside timestamps, are automatically transferred to the electronic prehospital medical record.

The study is reported according to the guideline Strengthening the reporting of observational studies in epidemiology (STROBE) statement [27].

### Selection of participants

We included patients over the age of 18 in the North Denmark Region to whom an ambulance was dispatched, and who had acute dyspnoea either according to DI at the emergency call, or as assessed by ambulance professionals on scene.

Patients were included in the period 1. July 2017 to 30. March 2019.

The study population included both patients transported to a hospital and patients treated and released on scene. All ambulance contacts were included in the study period.

### Variables

#### Dyspnoea scoring system

We used a verbal numerical scale design to assess dyspnoea, where the patient is asked, “*On a scale from 0 to 10, where 0 is no difficulties breathing at all and 10 is the worst possible breathlessness imaginable, how are you experiencing your breathing now?”* [22, 28] A similar phrasing is currently used by ambulance professionals in the North Denmark Region when assessing pain.

We implemented the dyspnoea scale as a registration field in the electronic prehospital medical record used in the ambulances. This enabled ambulance professionals to enter dyspnoea scale measurements directly into the electronic prehospital medical record. The dyspnoea scale module accepted numerical values 0–10 to be entered, and an automatic generated timestamp accompanied it. Ambulance professionals had two options when registering a patient as unable to use the dyspnoea scale, 1) due to the acute medical severity of the situation, and 2) other reasons, e.g. difficulty understanding the scale or language barriers.

Written instructions for use of the dyspnoea scale were provided to the ambulance professionals, who were instructed to carry out two dyspnoea scale measurements for each patient using the previously mentioned phrase. The first measurement was carried out at first contact with the patient, i.e. when the ambulance arrived at the scene. The last measurement was carried out at the last contact with patient, i.e. shortly before the ambulance arrived at a hospital, or prior to the patient being released on scene. The ambulance professionals treated the patients according to their usual guidelines [29].

#### Vital signs

As objective variables we included respiratory rate, blood oxygen saturation, blood pressure and heart rate. We obtained the values closest in time to the registered dyspnoea measurement. Clinically implausible vital sign measurements were excluded. The upper limits were set as 100 breaths per minute for respiratory rate, 300 mmHg for diastolic- and systolic blood pressure, and 300 beats per minute for heart rate.

### Analysis

We collected data on dispatched ambulances from the logistic dispatch system Logis CAD *(Logis Solutions A/S, Nærum, Denmark*). The patients’ prehospital measurements, including dyspnoea scores were obtained from the electronic prehospital medical record. We omitted missing vital sign measurements from their related analyses.

We used descriptive statistics when assessing the distribution of scores and measures of frequency. Mean and standard deviation was used for normal distributed data, and median and interquartile range (IQR) for non-normal. Normal distribution was assessed with histograms and quantile-quantile plots.

Inferential statistics with Wilcoxon matched-pairs signed-ranks tests were used to assess if the first measured dyspnoea scores differed from last measured. This was done for patients with two registered dyspnoea scores.

We used scatterplots to assess possible relations between dyspnoea score and vital signs and supplemented with linear regression analyses. This was done for the first measured score of patients with at least one dyspnoea score, and for the difference between first and last measurement, delta, for patients with two registered dyspnoea scores.

We assessed the feasibility of the dyspnoea scale as a tool in the prehospital setting, by investigating number of patients able to use the dyspnoea scale, patients’ pointwise change in score, and flooring- and ceiling effects. Furthermore, validity was assessed through the possible relations between dyspnoea score and vital signs.

Data were anonymised prior to analysis. Stata/MP 15.1 *(StataCorp LLC, Texas, United States of America)* were used for statistical analysis.

## Results

In the study period, 82,927 patients were seen by an ambulance professional. We included 3199 patients who had at least one registered dyspnoea score. Of these 2219 (69%) had two registered dyspnoea scores. Characteristics are seen in Table 1.

**Table 1:** Included patients

	Patients with at least one dyspnoea score	Patients with two dyspnoea scores
Number of patients	*3199*	*2219*
Age in years *(median, IQR)*	*74 (65 to 81)*	*73 (64 to 81)*
Female *(percent)*	*51*	*51*
Treat and release *(number, percent)*	*19 (0.6)*	*17 (0.8)*
Repeated user *(number, percent)*	*617 (19)*	*391 (18)*
Unable to use score *(number, percent)*	*673 (21)*	*122 (5)*
Acute medical severity	*306 (10)*	*76 (3)*
Other reason	*367 (11)*	*46 (2)*
Time between dyspnoea score and vital sign measurement in minutes *(median, IQR)*	*2.5 (0.9 to 6.5)*	*2.7 (1.0 to 7.0)*
Time to hospital in minutes *(median, IQR)*^a^	*44.9 (32.1 to 57.8)*	*45.0 (32.7 to 57.4)*
ICD-10 main chapter *(number, percent)*		
X Respiratory diseases	*1769 (55)*	*1270 (57)*
IX Circulatory diseases	*413 (13)*	*285 (13)*
XVIII Symptoms and signs	*327 (10)*	*224 (10)*

The characteristics of the included patients

*IQR* interquartile range, *ICD-10* International Statistical Classification of Diseases and Related Health Problems 10th Revision [30]

^a^Time from ambulance arrival at patient to arrival at hospital

Missing respiratory rate, blood oxygen saturation, diastolic- and systolic blood pressure and heart rate measurements were found for 126 (4%), 35 (1%), 79 (3%), and 35 (1%) patients respectively. Respiratory rate was measured automatically for 25% of the patients, and manually for the remaining 75%. No clinically implausible vital signs were found.

The 3199 patients with at least one registered dyspnoea score, had an initial dyspnoea score of a median of 8 (IQR 6–10).

**Fig. 1:**
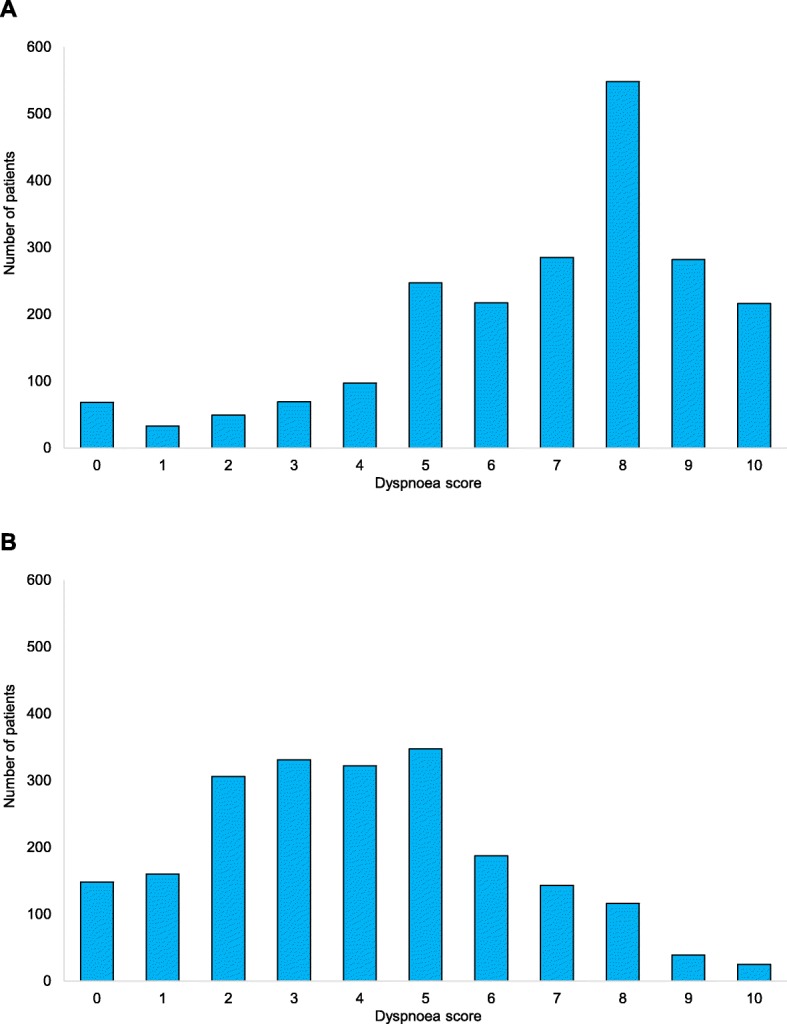
Dyspnoea scores. Distribution of dyspnoea scores for the patients with two measured dyspnoea scores. **a** Distribution of first measured dyspnoea scores. **b** Distribution of last measured dyspnoea scores. *N* = 2097 (patients unable to use score excluded)

For the 2219 patients with two registered dyspnoea scores, the first and last dyspnoea scores were statistically significant different (Wilcoxon matched-pairs signed-ranks test, *p* < 0.01). For 1676 (76%) of the patients, the first score decreased during prehospital treatment - from 8 (6–9) to 4 (2–5). The score was unchanged for 370 (17%) patients (2–8) and increased for 51 (2%) patients (from 5 (3–6) to 7 (5–8) (Fig. 1).

**Fig. 2:**
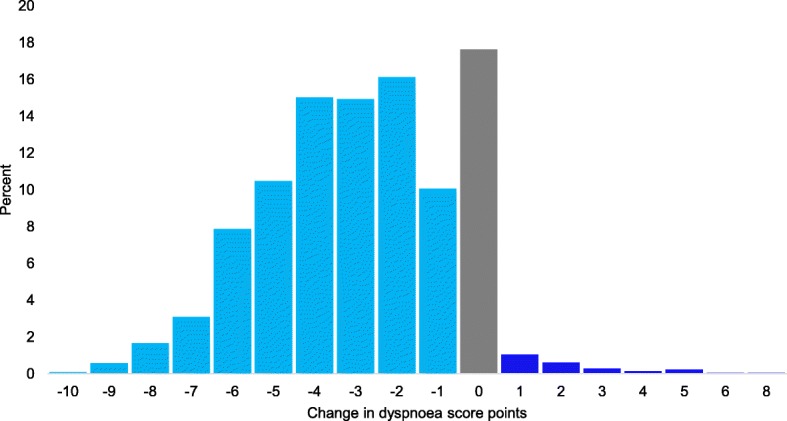
Change in scores. The individual pointwise change in score from the first- to last measured dyspnoea score. *N* = 2097 (patients unable to use score excluded)

For the patients who had a change in dyspnoea score, the individual patients’ pointwise change in score was 3 (2–5), with two, three, and four points being the most frequent (Fig. 2). The dyspnoea scale scores were distributed across the full range of the scale, with less than 9% of the scores at 0 and 10 (Fig. 1).

**Fig. 3:**
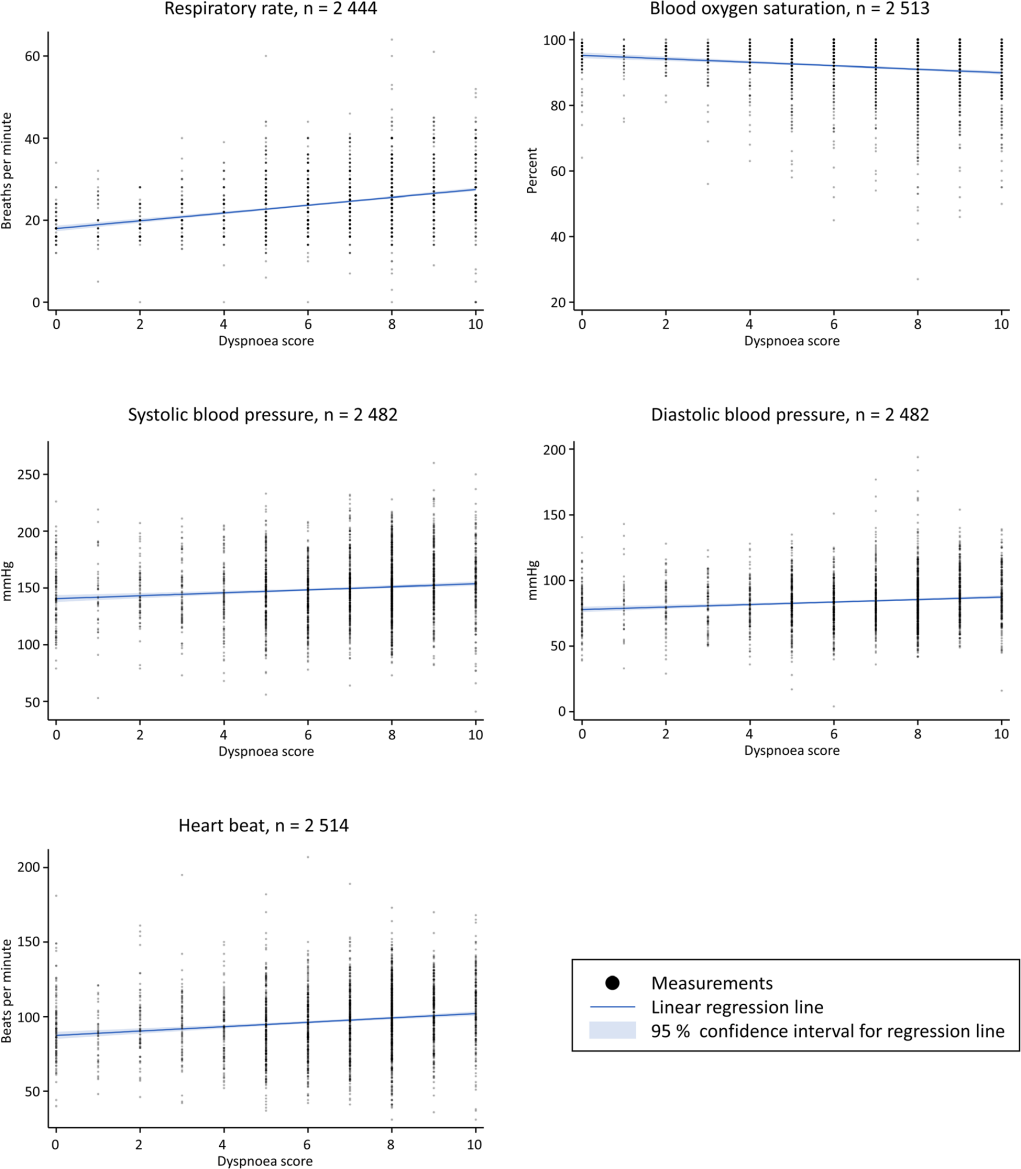
Dyspnoea score and vital signs. Scatterplots of the dyspnoea score and vital signs of the first measured dyspnoea score for patients with at least one dyspnoea measurement

For the first measured dyspnoea score of patients with at least one score, a statistically significant higher respiratory rate (linear regression, *p* < 0.01), diastolic- and systolic blood pressure (*p* < 0.01 and < 0.01), and heart rate (*p* < 0.01) was seen with higher dyspnoea scores (Fig. 3 and Table 2). In contrast, blood oxygen saturation was lower (*p* < 0.01) (Fig. 3 and Table 2).

**Table 2:** Linear regression slopes

Vital signs	Slope of linear regression line (95% CI)	
**First measured dyspnoea score and vital signs*(Patients with at least one dyspnoea score)***		
Respiratory rate	0.95	(0.86 to 1.05)
Blood oxygen saturation	−0.52	(− 0.64 to − 0.39)
Systolic blood pressure	1.3	(0.87 to 1.74)
Diastolic blood pressure	0.95	(0.66 to 1.24)
Heart rate	1.47	(1.13 to 1.81)
**Delta dyspnoea score and delta vital signs*(Patients with two registered dyspnoea scores)***		
Respiratory rate	0.45	(0.35 to 0.56)
Blood oxygen saturation	−0.37	(−0.52 to −0.22)
Systolic blood pressure	1.25	(0.83 to 1.67)
Diastolic blood pressure	0.38	(0.03 to 0.73)
Heart rate	0.28	(−0.02 to 0.59)

The slopes of the linear regression lines for first measured dyspnoea scores and vital signs. Furthermore, for the difference between first and last measurements i.e. delta scores and delta vital signs

*95% CI* 95% Confidence Interval

**Fig. 4:**
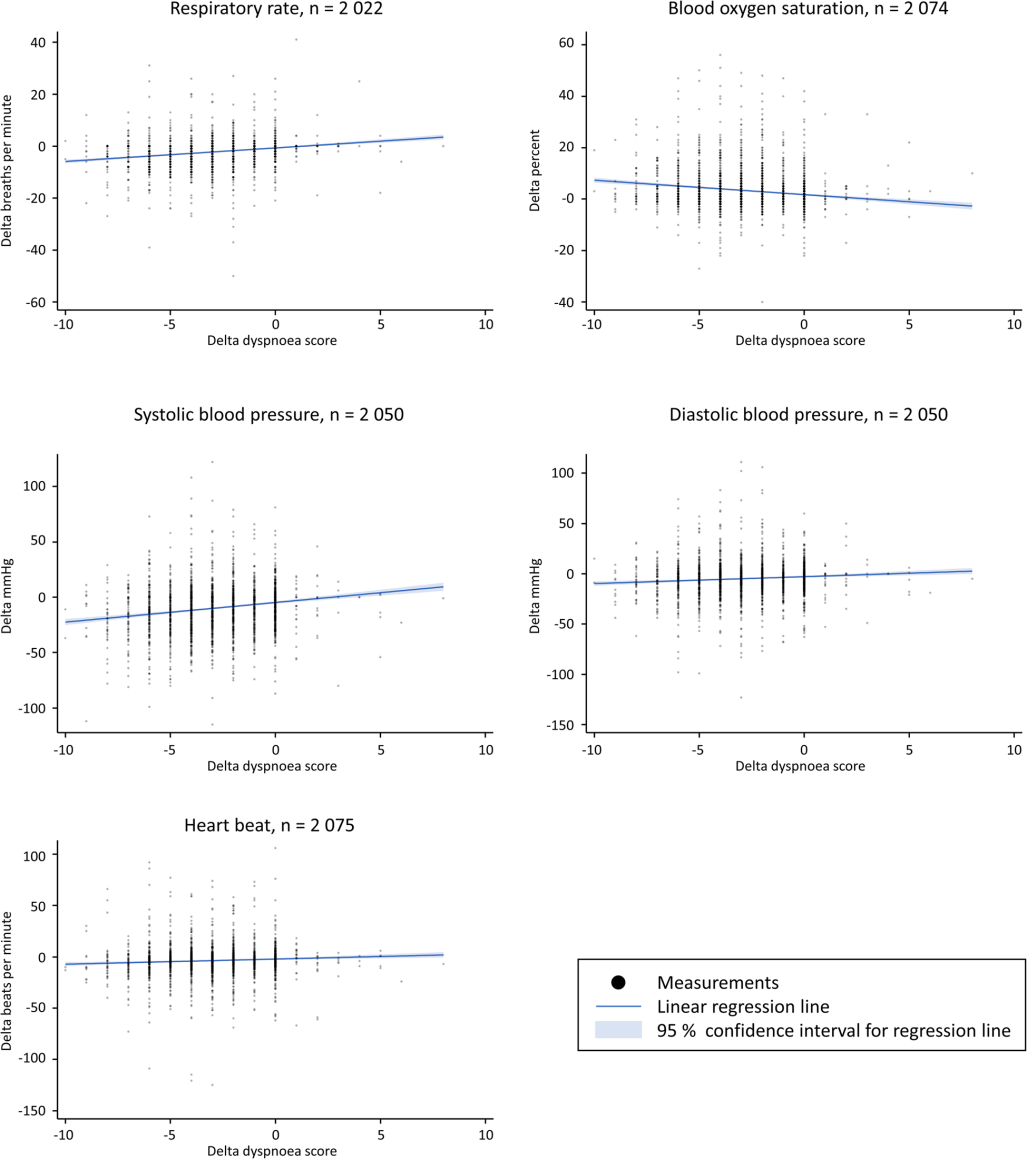
Dyspnoea score and vital signs. Scatterplots of the dyspnoea score and vital signs of the difference between first and last measurement (delta) for patients with two registered dyspnoea scores

For patients with two registered dyspnoea scores, statistically significant higher delta respiratory rate (*p* < 0.01), and delta diastolic- and systolic blood pressure (*p* < 0.01 and *p* = 0.03) was seen with higher dyspnoea scores (Fig. 4 and Table 2). Likewise, delta heart rate was higher (*p* = 0.07) and delta blood oxygen saturation was statistically significant lower with higher dyspnoea scores (*p* < 0.01) (Fig. 4 and Table 2).

## Discussion

In this study, we found that patients with acute dyspnoea had an initial high verbal dyspnoea rating score of a median of 8. This high value generally decreased during prehospital treatment. Respiratory rate, diastolic- and systolic blood pressure, and heart rate, were positively associated with increasing dyspnoea scores. Blood oxygen saturation was inversely associated with verbal dyspnoea rating scores.

We found ambulance patients had an initial high dyspnoea score, emphasising the severity of the symptom among ambulance patients. Like our prehospital study, other in-hospital studies found similarly high dyspnoea scores. A French study including 117 patients admitted to an emergency department with shortness of breath as their primary complaint [21]. The study assessed dyspnoea using both a verbal numerical- and visual scale, and found the patients had a median verbal numerical score of 7 (5–8) at admittance. An Australian study used a verbal dyspnoea score to assess dyspnoea for patients presenting with acute shortness of breath as either primary or secondary complaint in an emergency department [12]. The 253 included patients found a slightly lower median dyspnoea score, 6 (3–8), than found in our study. Different inclusion criteria and possible regional differences regarding emergency department patients, may explain the discrepancies in initial dyspnoea scores. Our study had a large sample size including patients from both urban and rural environments with a broader definition of dyspnoea, in contrast to the single emergency departments in both of the other studies [12, 21].

In our study, we found 76% of the patients had a decrease in score during the prehospital treatment. This suggest patients experience relief during the prehospital treatment, but it is uncertain if this is due to medical treatment, comfort, other factors or a combination of factors. Saracino et al. found a similar decrease from the dyspnoea score at initial triage and 30 min later [12]. The consistency with decreasing dyspnoea scores indicate the dyspnoea scale’s ability to report changes in experienced dyspnoea.

We found a statistically significant linear relation between the dyspnoea scores and vital signs measured in the ambulance. This relation was further strengthened by analysing both the initial dyspnoea score and vital sign measurements, and the difference between the first and last measurements, thereby assessing the relation between changes in dyspnoea score and vital signs. In addition, the relation between dyspnoea scores and vital signs were strengthened by the automatic measurements of vital signs, albeit 75% of respiratory rate measurements were counted manually. We had a high data completeness with the highest percentage of missing measurements not exceeding 4%. Finally, the ability to obtain the specific vital sign values documented as closest in time to the registered dyspnoea measurement was likewise a strength of the study. Most of the included vital signs were automatically measured and registered in the ambulance. They are the best indication, we have available for the patient’s acute status close to the time of the dyspnoea scale measurement, but measurement errors and equipment inaccuracies may be present. A study from the same region investigated vital signs measured in the ambulance in the period 2007–2014 and 2016, comprising 253,169 measurements, and found that less than 0.5% of vital signs were determined as extreme outliers /clinically implausible [31]. In our present study we also had a large number of measurements with no vital signs excluded as outliers. The errors and inaccuracies in the current study are therefore expected to have limited influence on our results.

Although statistically significant, the linear relation between dyspnoea scores and vital signs were of limited clinical relevance, when interpreting the slope of the linear relation line. The limited relation stresses the point of the patients’ experienced symptom assessed by the use of the dyspnoea scale, as the vital sign also do not reflect the degree of dyspnoea.

As our study, Saracino et al. found the verbal dyspnoea score to be significantly correlated to the patients’ respiratory rate, heart rate, and oxygen saturation, both at the initial measurement and 30 min later, which support our findings [12]. A study from the US included dyspnoea patients with a history of asthma or chronic obstructive lung disease in emergency departments and urgent care clinics [13]. It used a modified Borg scale at triage and 30 min following treatments. For patients with asthma, oxygen saturation decreased as the modified Borg scale score increased, however no correlation was found for chronic obstructive lung disease patients. In contrast our study used broader inclusion criteria and verbal numerical rating scale, which may account for the different correlations.

In general, the patients were able to use the dyspnoea scale, and they used the full range of the scale. The patients’ pointwise change in dyspnoea scores between the two measurements was nuanced with most frequently a change of two, three, and four points, rather than only the extremes i.e. 0 and 10. Less than 9% of the patients scored 0 or 10 at the two measurements. A previous study sat flooring- and ceiling effects limits at 15% as a validation criterion of acute respiratory scores, which our study honours [18]. Combined, these findings suggest the dyspnoea scale appear feasible to use, also in the prehospital setting. An alternative to the simple dyspnoea scale used in the current study could be health care professionals’ clinical assessment of the patient. In a Norwegian study both patients and healthcare professionals were asked to rate the patients’ dyspnoea severity on an 11-point numeric scale [14]. The study found the patients themselves reported higher dyspnoea scores than the nurses and physicians perceived they experienced. Likewise, a study from the United States of America also had patients and health care professionals assess the patients’ perceived dyspnoea, and found poor agreement between the two [15]. These studies emphasise the need for the patient’s own assessment, dyspnoea is a devastating symptom and the ability to detect relief is essential. In this regard, numerical ratings scales have previously been recommended a patient-reported outcome measure to assess severity of symptoms [32]. Combined with our assessment of the patients‘ ability to use the dyspnoea scale, it may be useful for obtaining patient-reported outcome measure of acute dyspnoea in the ambulance.

### Limitations

The primary limitation of the study is that we cannot report to which extent all acute dyspnoea patients were included, as this was decided over the phone at the emergency call or on the scene by the ambulance professionals. Even though the assessment carried out over the phone indicated dyspnoea as the main cause for calling, the patient’s actual medical condition may differ when ambulance professionals arrive at the scene, which can have led to patients being excluded from the study. However, the distribution of diagnoses according to ICD-10 main chapters were similar in this study as the findings in our previous study of the diagnostic pattern of patients with breathing difficulty [33]. Still, the methodological set up of this study was designed around the real life acute prehospital setting, and we must assume our findings can be generalised in similar settings.

The acute dyspnoea scale has the same limitation as many other subjective symptom assessment tools in regard to reliability and validity. We chose a simple verbal numerical rating scale rather than tools combining scales and questionnaires/verbal descriptors, due to the acute setting where simplicity and quick assessment is needed. The acute score in itself limits the options of testing the dyspnoea scales reliability, as the patient’s symptom may vary within short times, and it would not be possible to tell whether a difference between two scores were due to the acute diseases or the treatment or the scale itself. In regard to validity, the relation to the objective measured vital signs (increase in blood pressure, heart rate and respiratory rate, but decrease in blood oxygen saturation with increasing dyspnoea scores) indicate a certain construct validity.

Our study included patients with only a single dyspnoea scale measurement, and patients with at least two dyspnoea scale measurements. A higher percentage of patients unable to use the dyspnoea scale were found among patients with only a single dyspnoea scale measurement, compared to patients with at least two dyspnoea scale measurements. The patients with only a single dyspnoea scale measurement might be in a more severe condition making them unable to use the score. However, we found the two groups had similar characteristics, indicating the patients with only one score might not be in a more severe acute situation comparted to those with two scores. Yet, the patients with only one dyspnoea score present a limitation, as we were unable to determine if the patients had a worsening/improvement of symptoms during prehospital treatment.

## Conclusions

Patients with acute dyspnoea have initial high dyspnoea scores, which emphasises the perceived severity of the symptom, and the decrease in scores suggest the patients experience a relief of symptom during prehospital treatment. Acute dyspnoea scores were statistically associated with vital signs, but of limited clinical relevance. This stresses the importance of obtaining the patients’ experience of symptoms. To this end, we find it is feasible to use the dyspnoea scale in the prehospital setting.

## Data Availability

As the study include sensitive patient information, restrictions apply to the availability of data that is not publicly available. However, researchers interested in the data can seek approval from the Danish Patient Safety Authority. Having obtained approval, researchers can request data from the Centre for Prehospital and Emergency Care, Aalborg Denmark.

## Abbreviations

DI: Danish Index for Emergency Care
ICD-10: International Statistical Classification of Diseases and Related Health Problems 10th Revision
IQR: Interquartile range
